# Ninth International Medical Congress

**Published:** 1886-02

**Authors:** 


### Ninth International Medical Congress—Progress of Organization.

Two or three of our exchanges still repeat the assertion that no material progress has yet been made in the organization of the Congress. This is far from being true. On the contrary, the Preliminary Organization is at this time nearly complete. *First,* notwithstanding all that has been said about *declinations*, a large number of those appointed and published by the Committee on Organization, as first constituted, still hold their places, and for every one who has declined two equally well quailfied have cheerfully accepted positions. *Second*, the only important vacancies now existing are in the office of President of the Sections of Physiology, Pathology, and Gynecology, the filling of which has been delayed by the Executive Committee for reasons we have stated in previous issues of this *Journal.*

Not only is the *personnel* of the Organization thus nearly complete, but the proper officers are actively engaged in arranging the work for each Section, and the Executive Committee will doubtless be ready to issue a supplementary circular containing a full programme, early in May next. Already notices of contributions for several of the Sections have been received from prominent members of the profession in Great Britain, and indications favorable for a full European attendance are daily increasing.—*Journal American Medical Assaciation.*

